# An ‘explosion in the mouth’: The oral health experiences of autistic children

**DOI:** 10.1177/13623613241288628

**Published:** 2024-11-08

**Authors:** Amrit Chauhan, Kathy Leadbitter, Kara A Gray-Burrows, Karen Vinall-Collier, Nicola Pickles, Sarah R Baker, Zoe Marshman, Peter F Day

**Affiliations:** 1 University of Leeds, UK; 2 The University of Manchester, UK; 3 Airedale and Wharfedale Autism Resource, UK; 4 University of Sheffield, UK; 5 Community Dental Service, Bradford District Care NHS Foundation Trust, Bradford, UK

**Keywords:** autism spectrum disorders, education services, health services, interventions – psychosocial/behavioural, pre-school children, qualitative research, school-age children

## Abstract

In England, a quarter of 5-year-olds have tooth decay. For families with autistic children, establishing optimal habits (toothbrushing twice a day with fluoride toothpaste, limiting sugar intake and regular dental visits) can be difficult. Oral health experiences from the perspective of autistic children remains unexplored. Interviewing autistic children is crucial as it provides direct insights into their unique experiences and needs, ensuring their voices are represented in research. Therefore, we aimed to explore autistic children’s oral health experiences, including the barriers and facilitators to optimal oral health behaviours. We sought the views of 10 autistic children (aged 7–13 years old) about toothbrushing, healthy eating and visiting the dentist, using spoken interviews and Talking Mats^®^. Data were analysed using reflexive thematic analysis. Two themes were identified: (1) The diverse sensory nature of oral health activities; and (2) developing trust and routine through consistency, communication and collaboration. Our inclusive methodologies enabled autistic children with diverse communication needs to participate. Such methodologies improve the representation of autistic children in oral health research and autism-related studies more generally. The findings are informing the development of a new personalised support package to help families meet their autistic children’s unique needs.

**Lay abstract:**

In England, one in four children have tooth decay by the age of 5 years. Tooth decay affects many autistic children. Communication differences, sensory sensitivities and preferred routines can make dental care difficult. Daily toothbrushing, healthy eating and drinking, and attending the dentist may be challenging for autistic children. We do not know much about how autistic children feel about looking after their teeth. Learning from them directly is important to understand their needs and make sure their voices are heard. We interviewed 10 autistic children aged between 7 and 13 years to discover how they care for their teeth, what helped and what did not. We talked about toothbrushing, healthy eating and drinking and visiting the dentist. To support our conversations, we used Talking Mats^®^ – a tool that can help with communication. Autistic children described a wide range of sensory issues related to looking after their teeth. This finding shows how important it is to tailor care to each child’s needs. Children wanted to be included in conversations about their teeth at home and at the dentist. This was felt to make a big difference in building trust and making them feel comfortable and supported. Overall, we found Talking Mats^®^ can be used in dental research to engage with autistic children. By understanding children’s views, we can better help professionals and parents to support their dental needs. Our research showed that every child’s experience is unique, so dental support must be tailored and inclusive to meet children’s needs.

## Introduction

Tooth decay is a significant health problem affecting a quarter of 5-year-olds in England (GOV.UK, 2024). This figure rises to around 40% in more deprived areas (GOV.UK, 2024). Tooth decay directly impacts children’s quality of life, which, left untreated, causes toothache, sleepless nights and altered eating habits (Public Health England, 2017). Autistic children have similar levels of tooth decay to the wider childhood population but are less likely to visit the dentist and are twice as likely to need their dental treatment provided under a general anaesthetic (Sherriff et al., 2023). In England, treatment of decay is the most common reason young children (over 33,000 per annum) are admitted to hospital, costing the National Health Service over £40 million a year (GOV.UK, 2024).

Tooth decay can be prevented with twice daily toothbrushing with fluoride toothpaste (under parental supervision), limiting sugary foods and drinks and regular dental attendance as recommended by national guidance (Public Health England, 2021). These collective behaviours are referred to as ‘optimal oral health habits’ (Bhatti et al., 2021). Establishing these habits from early childhood provides protection across the life course (Hall-Scullin et al., 2017). However, autistic children’s communication differences, sensory sensitivities and preferred behaviour patterns can make optimal oral health habits difficult for families to establish (Alshihri et al., 2020; Du et al., 2019; Erwin et al., 2021; Public Health England, 2019). The National Health Service (NHS, 2019) long-term plan and Public Health England (2019)guidance prioritises the improvement of the health and wellbeing of autistic children. Despite this, the Cochrane Oral Health Group (priority setting partnership) found insufficient evidence of effective interventions that support autistic children with toothbrushing (James Lind Alliance, 2018).

Wider literature focuses on the oral health status of autistic children and the adaptions made by dental teams to facilitate clinical care (Du et al., 2019; Lewis et al., 2015; Thomas et al., 2018). However, a recent systematic review identified the absence of research into the lived experiences of autistic children of establishing and maintaining optimal oral health habits (Erwin et al., 2022). Struggles with daily oral care routines and accessing appropriate dental care are exclusively narrated by caregivers or dental professionals (Alshihri et al., 2020, 2021; Floríndez et al., 2021; Junnarkar et al., 2023; Parry et al., 2023; Parry & Shepherd, 2018; Stein Duker et al., 2019; Teste et al., 2021). Common themes across these studies demonstrate that families consistently report struggles with daily oral care routines, sensory discomfort during dental visits, and challenges in navigating the often-complex maze of access to dental care. Fenning et al. (2023) highlighted the effectiveness of a 1:1 therapist-led parent training intervention, delivered over 8–10 hours, in improving children’s oral health in the home and dental setting, underscoring the potential of caregiver-focused interventions. However, there is a critical gap in understanding the perspectives of autistic children themselves regarding their oral health.

There is an increasing recognition of the importance of engaging children as active participants to understand their perspectives and oral health experiences (Marshman et al., 2015). Understanding the oral health needs and preferences of autistic children allows the development of more person-centred, responsive and accommodating interventions, enhancing their acceptability, engagement and effectiveness. Recent developments in autism research underscore the importance of understanding first-hand perspectives and internal experiences (Courchesne et al., 2022; Leadbitter et al., 2021). Therefore, inclusive research methodologies that are accessible to autistic individuals with diverse communication needs and styles are essential. This includes those who are fully or partially non-speaking, uncomfortable communicating with unfamiliar people or stressed by demands (Cascio et al., 2021; Richards & Crane, 2020). Augmented and alternative communication (AAC) tools are particularly valuable in this context.

AAC tools can provide structure to a conversation, offer visual information to support or replace linguistic or auditory information and enable response options that are not reliant on spoken or written language (Iacono et al., 2016). There is growing evidence to support the use of AAC with autistic children (Iacono et al., 2016). Talking Mats^®^ is a visual communication tool that uses visual symbols to structure conversations, facilitating a more comfortable expression of preferences and experiences by autistic individuals (Hayden et al., 2024; Mundt, 2021; Stans et al., 2019). Research has shown that Talking Mats^®^ can effectively support communication for those with communication difficulties, improving their ability to participate actively in discussions about their needs and preferences (Coakes & Murphy, 2006; Murphy & Cameron, 2008; Stans et al., 2019). Incorporating such tools can ensure that the views of autistic children – and not just those able to participate in a spoken conversation – are heard and valued, aligning with the growing recognition of their right to actively participate in research. Therefore, we aimed to explore autistic children’s views on their oral health, including the barriers and facilitators to optimal oral health behaviours.

## Methods

### toothPASTE intervention design and a brief summary of the wider research

This study is part of a larger project funded by the UK National Institute for Health and Care Research called toothPASTE. toothPASTE aims to co-design an intervention that helps empower families to be more confident in looking after their autistic child’s teeth and establish optimal oral health habits. The research involved a scoping review, interviews with autistic children (n = 10), families of autistic children (n = 14) and professionals who care for them (n = 30) to capture the barriers and facilitators, what oral health support is available and what support is needed (ISRCTN16800746). The findings from the study reported here informed the co-design of the oral health intervention called toothPASTE. We worked with our lived experience advisory panel, professionals and stakeholders, to co-design the toothPASTE intervention. The qualitative interviews with families, professionals and the co-design of the toothPASTE intervention will be reported in separate articles. Ethical approval was obtained from the University of Leeds Dental Research Ethics Committee (DREC reference: 081221/PD/339).

#### Community involvement

An advisory panel of six parents with lived experience, such as having an autistic child, being autistic themselves and having an autistic child, or as a parent advocate for an autism charity, were actively involved in all stages of this research (including this work). The panel provided feedback on the accessibility and appropriateness of the recruitment poster, topic guides, Talking Mats^®^ symbols information sheets, consent/assent forms, and design of the interviews with autistic children. The panel lead (N.P.) co-wrote the academic article.

To develop the Talking Mats^®^ symbols, we consulted two teachers who had experience working with autistic children in mainstream and specialist schools.

#### Recruitment and sample

Children were enrolled in the study by their parents. We worked with key contacts within local and national autism charities, our advisory panel, schools and stakeholders to facilitate recruitment. A poster and information sheet were shared with parents by the designated key contacts using parents’ usual preferred communication methods, such as social media posts, newsletters, emails and letters. Interested parents were encouraged to contact the research team via email or phone. Recruiting through a range of organisations ensured that a diverse cohort of children across different ages, locations, educational backgrounds and ethnicities took part (purposive). Following parental informed consent, interviews were arranged at convenient times for the participants, either at home or school. Where possible, written child assent was collected on the day of the interview. Where this was not possible, child assent was assessed through their willing engagement and participation.

### Participants

Overall, 12 children were recruited for the study. However, two were excluded, resulting in a final sample of 10 children. These 10 autistic children met the following eligibility criteria: (1) aged between 5 and 14 years old, (2) confirmed or working diagnosis of autism, (3) located within an hour’s drive from collaborating centres and (4) able to communicate views related to the research questions at least to some extent, either verbally or non-verbally.

Prior to the interviews, time was taken to build rapport and assess each child’s ability to communicate their views. This informal assessment was based on our interpretation of the children’s responses to our engagement efforts either verbally or non-verbally. Despite these careful preparations, two children found it difficult to engage with the research questions. It became evident that these children could not express their views due to their level of symbolic understanding, despite adaptations to accommodate their needs. This was further corroborated through discussions with their parents. As such, they were excluded from the results but are featured in the demographic table to provide an overview of the sample.

### Theoretical framework and methodological orientation

This research is grounded in a realist epistemology, which assumes a direct relationship between language and the experiences it describes (Willig & Rogers, 2017). The methodological approach aligns with the flexibility of reflexive thematic analysis by focusing on both explicit meanings and underlying patterns in participants’ accounts (Braun & Clarke, 2006, 2021). This prioritises children’s narratives about their attitudes and experiences regarding oral health, ensuring their perspectives are accurately captured through both latent and semantic coding. The realist framework balances the clarity of semantic coding with the deeper insights from latent coding, maintaining methodological rigour and philosophical coherence in line with qualitative research (Denzin & Lincoln, 2011; Willig & Rogers, 2017).

### Data collection

The interviews were conducted by a team of female researchers from the disciplines of Psychology and Dentistry, including A.C. (Project Manager and lead Qualitative Researcher, CPsychol, PhD, BSc), K.L. (Research Fellow, PhD, BSc) and a dental researcher (BSc). Each interview was conducted by a lead researcher (A.C. or K.L.) with support from a note-taker/photographer (A.C., K.L., or dental researcher). Before the interviews, A.C. spoke with the parents/teaching assistant to learn about the child and their communication preferences. All interviews were face-to-face. Interviews began with rapport-building, as the researchers were not known to participants. A familiar adult (parent/teaching assistant) was present throughout to support the child’s wellbeing and participation. The researchers ensured that responses were the child’s own and not influenced by their familiar adult. A broad topic guide was used and adapted for Talking Mats^®^, ensuring tailored communication support (see Supplementary materials). The interviews were audio recorded and photographs taken of the Talking Mats^®^. Interviews were professionally transcribed. Interviews lasted 40–60 min, with children receiving a £10 Amazon voucher. Transcripts were not provided to participants. Debriefing sessions and reflective notes post-interview facilitated the team’s reflexivity, data interpretation and modifications to data collection.

#### Talking Mats^®^ to facilitate interviews

**Figure 1.**
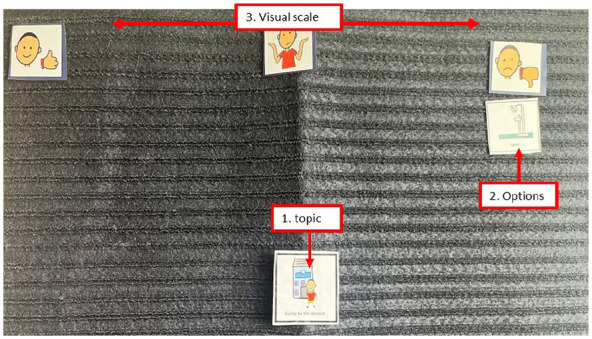
An example of Talking Mats^®^ with (1) the topic being explored – visiting the dentist; (2) options about the topic- light; and (3) a visual scale – ‘like’ on the left, ‘not sure’ in the middle and ‘not like’ on the right. The options (2) would be introduced and placed under the visual scale items that best reflect their preference. In this example, the light has been placed under the ‘not like’ section.

Talking Mats^®^ is a communication tool used to support individuals, including autistic children, to express their thoughts, feelings and experiences (Mundt, 2021; Talking Mats, 2013). Talking Mats^®^ uses a structured and simple visual framework consisting of a textured mat and three sets of picture symbols (see Figure 1): (1) the ‘topic’ being explored, (2) ‘options’ about the topic and (3) a ‘visual scale’ (Talking Mats, 2013).

The visual scale consisted of ‘like’, ‘dislike’ and ‘not sure’. Children were presented with a set of picture symbols (options) about the topic, which included ‘Toothbrushing’, ‘Going to the dentist’ and ‘Things I eat and drink’. One-by-one the options were handed to the child, with the question, ‘How do you feel about . . . ?’. Children placed option symbols on the mat next to the visual scale to indicate their response. Children were asked follow-up questions verbally (e.g. ‘Can you tell me more about . . . ’) where they were perceived to be comfortable with this approach. Children could also choose to respond through a simple placement of the picture without speaking and were not pressed to respond verbally if they were perceived to be uncomfortable or unable to do so. Following Talking Mats^®^ principles, children were able to draw their own ‘options’ to include on the mat.

Before the interviews, A.C. spoke with the parents or teaching assistants to learn about the child’s interests and their communication preferences. Where possible, Talking Mats^®^ was used as a standardised approach; however, this was contingent on the child’s preferences. Talking Mats^®^ was chosen for its adaptability to various communication styles and needs, facilitating a structured yet flexible way for children to express their thoughts and feelings during the interviews (Talking Mats, 2013). This approach ensured that while a consistent method was employed, it was tailored to accommodate individual communication preferences identified in preliminary discussions with parents. If a child demonstrated, or parents reported, a clear preference for other forms of communication (e.g. verbal only, use of props), those methods were pursued instead. For example, if a child chose to communicate by showing their own toothpaste or drawing a toothbrush, this was welcomed and documented. Equally, if the parent stated that the child would prefer to be asked questions without using Talking Mats^®^, this was adopted.

### Analysis

Data were analysed using reflexive thematic analysis (Braun & Clarke, 2006, 2021) and adapted to integrate the Talking Mats^®^ data. An inductive approach was undertaken, using semantic and latent coding. Semantic refers to analysing the explicit content, whereas latent goes beyond this to explore the assumptions underlying the data (Byrne, 2022). The analysis included (1) familiarisation with the dataset, in which A.C. listened to the audio recordings, anonymised the transcripts, re-read and made initial notes of the interviews and Talking Mats^®^ data; (2) generating codes at a semantic and latent level, capturing significant features and patterns; (3) the construction of initial themes by clustering codes that shared similar meanings or concepts derived from the data; (4) the development and review of themes to ensure they reflect the dataset and how they relate to each other; (5) refinement, defining and naming the themes to ensure each theme was distinct; and (6) write up. The supplementary material provides a detailed account of the iterative development of themes and codes, including the final coding output, which presents the themes, subthemes and example codes developed from the analysis. Data were organised, analysed and managed using NVivo version 14 (Lumivero, 2023).

For verbal responses during stage 2, codes were developed directly from the transcripts by A.C. Simultaneously, Talking Mats^®^ data were interpreted with simple descriptions from the photographs such as ‘did not like going to the dentist’ to summarise their placements. This facilitated an additional sense of scale and patterns within the data. During stage 3, the data from Talking Mats^®^ were mapped onto the initial themes and subthemes that emerged from the codes developed from the spoken interviews. This process ensured that the themes were grounded in the data collected from both verbal responses and Talking Mats^®^ interpretations. In addition to demonstrating the patterns, it provided an additional layer of context and support that complemented the identified themes. During stages 4–5, A.C. continuously reviewed the themes, so they accurately reflected the combined insights from all children and all communications, verbal or non-verbal, ensuring Talking Mats^®^ data were treated with equal rigour and consideration as spoken communication.

Themes were discussed with wider research team members (P.F.D. and K.A.G-B.). Analysis was an iterative process, where themes were developed and refined over time. Throughout the process, the authors from different disciplines (Dentistry, Dental Public Health, Mental Health and Psychology) were involved in sense checking. Initial themes were discussed with the advisory panel during a workshop meeting. The themes were presented in a written format, and A.C. described the content of each theme. The panel then discussed the themes among the group, providing comments and feedback based on their own experiences. Adjustments were made to the themes to ensure they closely aligned with the lived experiences. For example, the challenges of attending the dentist were discussed, including how ‘professionals need to get into the child’s world’. This feedback reinforced the trust and collaboration theme described below. Reflexive considerations are reported in the supplementary material.

### Trustworthiness and rigour

We employed several strategies to ensure our study’s trustworthiness and rigour. For example, we shared preliminary findings with our community involvement panel to confirm the reflectiveness of our interpretations and outlined the researcher’s own position within the analysis (see Supplementary materials). To maintain dependability, we kept detailed documentation of our research process, including field notes and reflections. Transferability was supported by providing detailed descriptions of the research context and participants (see recruitment and sample), enabling readers to assess the applicability of our findings to other settings. Confirmability was achieved through reflexive journaling, peer debriefing and incorporating feedback from our community involvement panel, mitigating potential researcher bias. Finally, the writing of this report was informed by the Consolidated Criteria for Reporting Qualitative Research (COREQ) guidelines to enhance transparency (Tong et al., 2007). These example strategies collectively ensured the robustness and validity of our study’s findings.

## Findings

Two overarching themes were developed: (1) The diverse sensory nature of oral health activities and (2) developing trust and routine through consistency, communication and collaboration. Each theme comprised of several subthemes as outlined in Table 1. Throughout, Talking Mats^®^ pictures are provided alongside quotes of spoken language, to provide evidence from participants who communicated non-verbally as well as those who communicated verbally.

**Table 1.** Themes and subthemes.

Theme	Subthemes
1. The diverse sensory nature of oral health activities	1a. Toothpaste can feel like an ‘explosion in the mouth’.
	1b. ‘Too soft’ or ‘too hard’ the importance of the right oral health tools and techniques
	1c. An overwhelming environment
2. Developing trust and routine through consistency, communication and collaboration	2a. Navigating the uncertainty of dental care: Continuity and transparency
	2b. Seeking autonomy and collaboration in oral health decisions
	2c. Parental role and support in creating oral health habits
	2d. Visual support for motivation and routine: helpful versus impractical

### Theme 1: the diverse sensory nature of oral health activities

The theme emphasised the uniqueness of each child’s sensory experience and the diversity of responses to different textures, tastes, pressures, lights, smells and sounds. The complexity of these sensory experiences was evident throughout the data, both at semantic and latent levels.

#### Toothpaste can feel like an ‘explosion in the mouth’

Some children described how some toothpaste felt like an ‘explosion’ in the mouth and used similar metaphorical descriptions:

I’m fine if it just goes on my teeth and I don’t like put my tongue on it by accident. But if it ever goes on my taste buds it just blows up. (Harry)

The descriptors of ‘explosions’, and ‘blows up’ evokes feelings of discomfort, pain and a potentially overwhelming experience. This could be interpreted thematically as a battle, where autistic children may have to prepare themselves for a strong unpleasant sensory overload when engaging in oral health activities. The metaphorical language used about oral health products and dental equipment highlights their potential as barriers to oral care. To overcome these, children discussed how they preferred toothpaste with a non-foaming flavourless profile:

I have a special toothpaste . . . it don’t taste like anything and it doesn’t bubble in your mouth because I hate when it, when it bubbles cause it looks disgusting. (Hannah)

**Figure 2.**
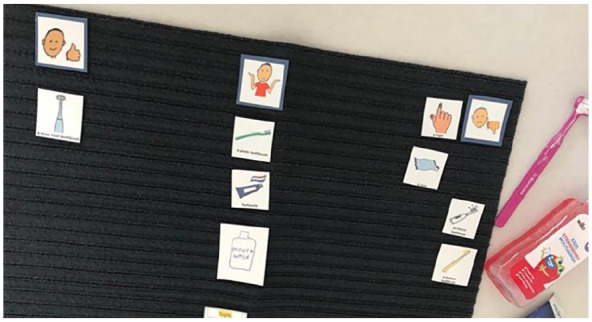
Image of Amber’s Talking Mats^®^ (what I use to clean my teeth). This represents the Talking Mats^®^ of Amber when asked about what she uses to clean her teeth (topic placed middle bottom). As can be seen, they disliked using their finger, a cloth, an electric toothbrush and bamboo toothbrush (right-hand side of the mat). They placed the plastic toothbrush, toothpaste and the mouthwash in the ‘not sure’ section (middle of the mat). They put the three head toothbrush in the ‘like’ section (left of the mat). The ‘mouth wash’ was an additional ‘option’ that Amber wanted to include as she showed the researchers the mouthwash she used (see right of picture).

This ‘special toothpaste’, in comparison to a regular toothpaste, caters specifically to their individual sensory needs (the absence of strong mint taste and foam). The child’s use of the word ‘hate’ (often shared by other children, along with ‘dislike’ on the Talking Mats^®^) suggests an emotional reaction to the foaming element of the regular toothpaste. While some children leaned towards flavourless and non-foaming toothpaste, others preferred fruit-flavoured or milder mint-flavoured options, which aligned with their personal sensory preferences. For example, Amber showed the researchers their flavourless and non-foaming toothpaste (oraNurse). They had placed their Talking Mats^®^ symbol of ‘toothpaste’ in the middle of the mat (see Figure 2).

This reaction led the researchers to ask ‘What would make a toothpaste really good for you? If you could have a magic toothpaste?:

Rainbow toothpaste that does not foam up at all with with . . . raspberry and cherry flavoured. (Amber)

Therefore, while the milder toothpaste was preferred, it could suggest that Amber still had not found the toothpaste that they liked using, which can be shared similarly by children such as Hannah, Ron and Lennie who placed the toothpaste symbol in the ‘not sure’ section. The visual quality was important, especially given that Hannah highlighted how their previous mint-flavoured toothpaste ‘looks’ disgusting. This preference signifies a multifaceted sensory engagement, where visual stimuli (rainbow), taste (fruit, mint or flavourless) and texture (non-foaming) all play a role in shaping children’s positive perception of the toothpaste they prefer.

#### ‘Too soft’ or ‘too hard’ the importance of the right oral health tools and techniques

The experiences with oral health tools, both at home and at the dentist, were frequently described as uncomfortable reinforcing the importance of the right tools. Children highlighted the significant impact of the textures, pressures and techniques of toothbrushes and dental equipment on their comfort levels. For example, the feeling of the dental equipment at the dentist was often described as uncomfortable and ‘weird’:

. . . they like put the thing in your mouth and the mirror and they just go scraping around and numbering the teeth. It just feels weird. (Harry)

There’s like this thing that goes into my mouth and there’s like a machine that scans my mouth and I don’t like it ’cause it makes weird beepy sounds (Amber)

They [dental equipment] feel weird on your teeth. (Ron)

This ‘weirdness’ relates not only to the feeling of pressure of the dental equipment on the teeth but also to the auditory experience of ‘weird beepy sounds’ that may be both unfamiliar, unsettling and an out-of-the-ordinary sensory expectation. Like dental equipment, electric toothbrushes were also portrayed as ‘too loud’, which may become an intrusive sensory element, causing discomfort. This auditory sensitivity aligns with the children’s perceptions of dental equipment and the ‘strange sounds’, revealing a consistent theme of sensory sensitivities across a wide range of oral health activities, thus reinforcing the importance of the right tools.

Other sensations (sounds, texture, taste and pressure) were also discussed:

Cause sometimes the bristles on [an electric toothbrush] doesn’t feel nice on my teeth (Ron)

Is that when it’s really hard or it’s really soft? (Interviewer)

When it’s really hard . . . I don’t like it soft either, I like it in-between (Ron)

The preference for an ‘in-between’ toothbrush that was not too hard or soft could indicate their need for a sensory output that is neither over- or under-sensitive. The variation highlights the individualised (and diverse) sensory needs of autistic children in relation to dental equipment and oral health products, particularly as comments concerning toothbrushes ranged widely, reflecting different preferences for electric, manual, three-sided, bamboo or plastic toothbrushes. For example, Ash, who drew a toothbrush, described how the material of the toothbrush (such as the composition and texture of the handle) was equally important. This further underscored the perceived importance of the right tools tailored to individual sensory needs:

Has it got wings or is it . . . a flying toothbrush? (Interviewer)

I have a wooden toothbrush, so number one, if you have a wooden toothbrush and it gets soaked in too much water and then you start brushing your teeth, you’ll get splinters (Ash)

**Figure 3.**
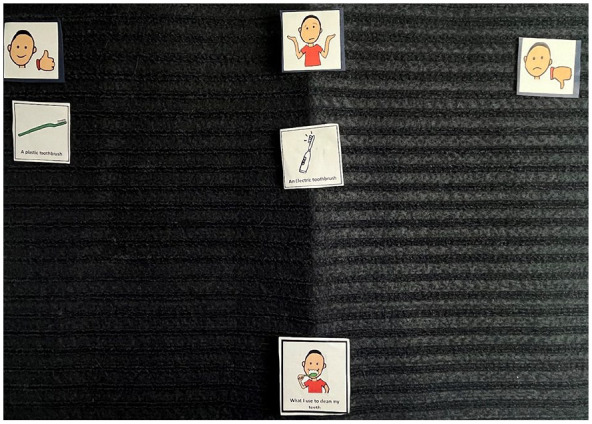
Image of Arun’s Talking Mats^®^ (what I use to clean my teeth). This represents the Talking Mats^®^ of Arun when asked about what he uses to clean his teeth (topic placed middle bottom). As can be seen, they placed the plastic toothbrush in the ‘like section’ (left of the mat) and electric toothbrush the ‘not sure’ section (middle of the mat).

**Figure 4.**
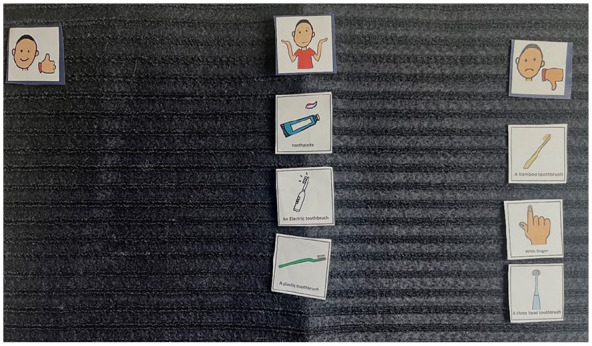
Image of Ron’s Talking Mats^®^ (what I use to clean my teeth). This represents the Talking Mats^®^ of Ron when asked about what he uses to clean his teeth. As shown, they did ‘not like’ the bamboo toothbrush, finger and three-headed toothbrush (right-hand side of the mat). They placed the toothpaste, electric toothbrush and plastic toothbrush in the ‘not sure’ section (middle of the mat). Nothing was placed in the ‘like’ section (left of the mat).

**Figure 5.**
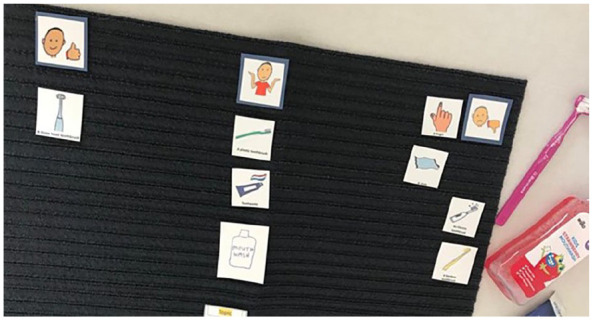
Image of Summer’s Talking Mats^®^ (what I use to clean my teeth). This represents the Talking Mats^®^ of Summer when asked about what she uses to clean her teeth (topic placed middle bottom). As can be seen, they did ‘not like’ using their finger, a cloth, an electric toothbrush and bamboo toothbrush (right-hand side of the mat). They placed the plastic toothbrush, toothpaste and the mouthwash in the ‘not sure’ section (middle of the mat). They put the three-headed toothbrush in the ‘like’ section (left of the mat). The ‘mouth wash’ was an additional ‘option’ that Summer wanted to include as she showed the researchers the mouthwash she used (see right of picture).

Talking Mats^®^ data suggested that although toothbrushes were generally disliked, some were more tolerable than others, with preferences varying among individuals. For instance, some children favoured electric toothbrushes, possibly indicating under-sensitivity, while others preferred toothbrushes with softer bristles, suggesting over-sensitivity (see Figures 3 to 5).

These preferences highlight the heterogeneity in sensory experiences among the children interviewed.

#### An overwhelming environment

**Figure 6.**
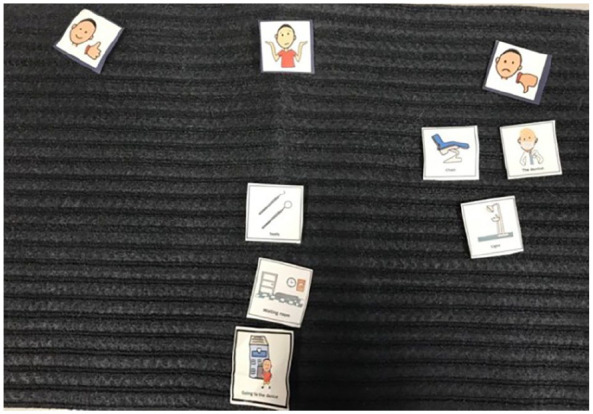
Image of Kai’s Talking Mats^®^ (going to the dentist). This represents the Talking Mats^®^ of Kai when asked about Going to the dentist. As can be seen, they did ‘not like’ the dental chair, the dentist and the lights used. They placed the dental tools and the dental waiting room under the ‘not sure’ section.

Children also communicated sensitivity to the broader environmental context in which oral health activities took place. They highlighted how noisy areas at home where they brush their teeth, the smells and noise when eating and the bright lights at the dentist contributed to their discomfort (see Figure 6).

I mean often I’m, I like close my eyes because it’s like it’s the fact that it can be quite bright and . . . often . . . when . . . they give me the sunglasses that they give me, often like when it goes back I start to close my eyes . . . . It’s like I know that it’s going to be really bright. (Ellie)

Is there a reason why you don’t like the lights? (Interviewer)

They’re too bright. (Lennie)

Does it help when you put glasses on? (Interviewer)

Not really. (Lennie)

During the ‘what I eat and drink’ topic, the children provided descriptions of their preferred and disliked foods and drinks (often indicating a preference for high sugar). Although unprompted, children often discussed their experiences of eating at school, focusing on the sensory challenges they faced in the dining hall. Their difficulties with overwhelming noise levels, crowded spaces and strong smells mirrored the sensory challenges they also encountered at the dentist:

It’s loud [the school dinner hall], it, and it smells and it’s not always too nice just to be in there. It’s like not really nice to smell what other people are eating. (Ellie)

Referring to the dinner hall as ‘loud’ and commenting on unpleasant smells highlights the complex, multi-sensory nature of environmental sensitivities. There could be hyper-awareness and heightened sensitivity to odours. These sensory experiences may interact in nuanced ways, shaping how autistic children perceive and engage with their surroundings. The aversion to smells during mealtime may reduce appetite, disrupt concentration on eating or even avoidance of certain foods. This was captured by Ellie’s experience where she stated her preference to eat in quieter areas of the school or avoid eating at all:

I normally take food with me, but I don’t always eat it. (Ellie)

### Theme 2: developing trust and routine through consistency, communication and collaboration

**Figure 7.**
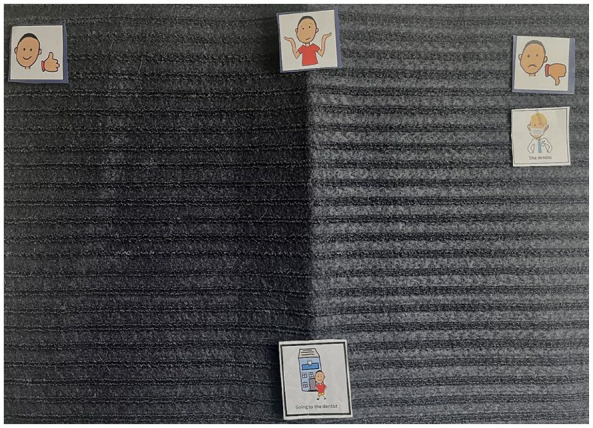
Image of Arun’s Talking Mats^®^ (going to the dentist). This represents the Talking Mats^®^ of Arun when asked about going to the dentist. As can be seen, they placed the dentist in the ‘not like’ section (right of mat).

This theme underscores the significance of a collaborative approach among children, parents and dental professionals in supporting and establishing optimal oral health behaviours. The data illustrate a nuanced relationship between dental professionals, children and their parents, all working together to form a personalised oral health care experience. However, children often reported negative experiences when attending the dentist (see Figure 7). This was clearly demonstrated when many children placed their Talking Mats^®^ symbol in the ‘not like’ area, indicating their clear discomfort and dissatisfaction:

#### Navigating the uncertainty of dental care: continuity and transparency

The dental clinic often emerged as a setting that led to unpredictability and worry, particularly around the change of dentists. Such inconsistency, while possibly unsettling for many children, can pose heightened challenges for those who often rely on routine and familiarity:

There seems to be a lot of different dentists. I don’t think I’ve ever seen the two same, the same dentists twice. (Harry)

And do you think it’d make a difference if you did see the same person? (Interviewer)

Yeah cause then I would know how they work (Harry)

In this exchange, Harry’s words articulate beyond a preference; they hint at a deeper need to anticipate and understand their dentist. Their suggestion for consistency is not just about comfort but establishing a sense of control in an environment that could feel overwhelmingly unfamiliar. The phrase ‘I would know how they work’ seems to suggest that not only do they want to understand the clinical procedures and how it will be delivered, but also build a rapport with the dental professional. This suggests that as well as familiarity, a mutual understanding can greatly enhance the comfort and predictability of the experience.

This sense of trust not only encapsulates familiarity but also transparency:

I prefer the ones that tell me what it’s going to be like. (Harry)

Like, oh no, no, I don’t, I don’t like that, no. Cause they don’t say a single thing about it either. It’s just like they want to shove horrible contra[p]tions in your gob [mouth] for no apparent reason. (Lennie)

The phrase ‘for no apparent reason’ conveys a lack of explanation provided by the dentist. This lack of communication resonated with an overarching theme in the data, where children felt uninformed and disconnected from their dentist, leading to a lack of trust. Furthermore, the vivid imagery of ‘horrible contra[p]tions’ being ‘shoved’ captures the child’s sensory reaction to the dental visit, highlighting not just the physical discomfort but also a distressing experience. This was heightened by the repetition of ‘no’ to express their clear discomfort.

#### Seeking autonomy and collaboration in oral health decisions

Children indicated not only a preference for consistent, clear communication but also a desire to be involved in the decision-making process:

I need a reason . . . .I need a reason to say yes or no to things. That’s, that’s also when I say I do or don’t like things. I just, I don’t just don’t like something, I normally have some kind of reason. (Lennie)

The need to voice their reasons not only highlights the importance of transparent dialogue, but also how they would like to be active participants in their care journey, not passive recipients. Such narratives suggest that the children interviewed would have liked an opportunity to allow their dentist to have an insight into their world and to be able to share what it is like for them, especially when they arise from specific preferences or sensitivities. This collaborative discussion has been demonstrated in Summer’s account when placing the toothpaste in the ‘not sure’ section of the Talking Mat^®^:

Oh gosh. If the toothpaste, if the toothpaste is a little bit too minty, then I don’t like it . . . . I’m using, like, a tasteless non-foaming one [toothpaste] . . . . The dentist recommended it to us, and it has been pretty helpful, hasn’t it, Mum? . . . He gave me stickers and he gave me a new toothbrush on my birthday sometimes. Apparently, you should change your toothbrush every three months though. (Summer)

The use of ‘us’ signifies the inclusion of both the child and her parent, highlighting a collaborative approach in the decision-making process. This collaboration is evident when the dentist recommends a specific toothpaste tailored to Summer’s sensory sensitivity and dislike to strong mint flavours, demonstrating an understanding and recognition of her individual needs. In addition, the inclusion of stickers and toothbrushes contributes to an engaging dental visit, allowing Summer to feel both included and actively involved. This involvement underscores the sense of autonomy and collaboration in oral health decisions.

#### Parental role and support in creating oral health habits

**Figure 8.**
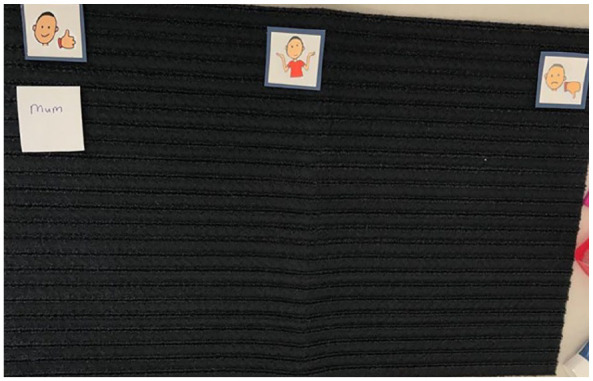
Image of Talking Mats (Amber). This represents the Talking Mats^®^ of Amber when asked about what she used to brush her teeth. This was an additional mat following this topic. She placed the option of ‘mum’ in the ‘like’ section (left of mat). The other sections were left blank (see middle and right of the mat).

The findings also highlighted the essential role and engagement that parents (or caregivers) have in supporting their children’s oral health behaviours. This was shown by Amber (see Figure 8) when they were asked who they liked to help them brush their teeth.

The child’s relationship with their dentist, although vital, was intertwined within the broader context of their home environment and parental support system. Some children, for example, described how they would often forget to brush their teeth when feeling tired or unmotivated:

Normally because I’m getting in bed soon and that’s annoying and I can’t be bothered to. Sometimes I don’t forget and sometimes it’s just my laziness but most a’ the time it’s when I forget. (Lennie)

I do it most days . . . . It’s usually because I’m in a rush or something. (Harry)

Parents actively take a role in shaping daily routines, offering reminders and encouragement to undertake these behaviours. For example, when children were asked how they were supported to improve their oral health, Ash replied, ‘*Just reminding me more often so I don’t forget to do it*’, signifying an acknowledgement of their parent’s role. This was not an isolated sentiment, as other children also described how their parents frequently asked whether they had brushed their teeth:

So often like sometimes its just like check-up, like see if I’ve forgotten or not. (Ellie)

The dynamic between motivation and parental support was further amplified, with parents stepping in to assist when their child felt unmotivated or reluctant to engage in oral care:

who brushes your teeth? (Inteviewer)

Sometimes I do. (Ron)

Hmmm very rare . . . (Mother)

If I want to I will. (Ron)

This interaction illustrates the complexities of developing consistent routines. The parent’s use of ‘very rare’ may indicate a greater underlying concern and implies that the parent may play a more active role in toothbrushing. The child’s statement captures a sense of autonomy and independence but also suggests a lack of consistent practice, indicating a disconnect between intention and implementation.

#### Visual support for motivation and routine: helpful versus impractical

Many children interviewed described how they used visual aids as effective tools, not only to remind them to brush, but also to make toothbrushing and visits to the dentist feel more engaging. Visual cues, such as charts or videos, were reported as particularly helpful. These serve dual purposes, as they helped toothbrushing feel quicker and acted as timely reminders:

Often in like holidays I do this sometimes because I know that it’ll just go by really fast if . . . I decide to watch something. And it’s probably what I most enjoy doing, yeah. – Lennie

But I just think I need to do that more often[use the whiteboard], cause it has brush teeth on it in the morning and in the night. (Summer)

And is it important to have, like, a little white board, and have a routine? (Interviewer)

Yeah. It’s like a calendar white board thing, but I use it to put, like, routines on it, so that I can get into the swing of stuff. (Summer)

One child described playing on a parent’s phone in the dentist’s waiting room, a strategy that alleviated pre-appointment nerves:

Sometimes I just ask if I play on mum’s phone and it sorta distracts me from being nervous. (Harry)

Just as visual schedules provide structure and predictability, playing on a phone offers a distraction that helps manage nervousness. This use of visual engagement highlights the broader concept that visual aids, whether digital or physical, play a crucial role in creating a calming routine and making dental visits more manageable and less intimidating for the children interviewed. Visual supports can be both practical and beneficial in facilitating a positive dental care experience. Furthermore, by allowing the child to use a familiar device, the parent shows understanding and provides a coping method; this illustrates a collaborative approach to alleviate the child’s worry.

However, the interviews also revealed instances where visual aids did not integrate with the children’s routines. For instance, some noted the difficulty in the practicality of using digital/electric visual aids such as an iPad while brushing teeth:

Well, can’t really do that because mum needs my head up, that means I’ve got to put the iPad like that [raises hands high above head]. (Amber)

While visual aids serve as invaluable tools for the children interviewed, their efficacy is contextual. Their applicability differs based on the situation and the individual needs of the child. For some, a visual aid such as an iPad or phone is the anchor that eases their nerves; for others, it adds further challenge to an already intricate process.

## Discussion

This research aimed to explore autistic children’s oral health practices and preferences. We developed two themes that capture the barriers and facilitators influencing optimal oral health behaviours. Within the field of dentistry, this is the first research in the United Kingdom where the views of autistic children have been actively sought, utilising Talking Mats^®^ to facilitate meaningful engagement and capture their perspectives.

The theme, ‘The diverse sensory nature of oral health activities’, reflects the multifaceted and individualised sensory experiences that autistic children encounter when managing their oral health. Although the sensory sensitivities of autistic children have been extensively documented (He et al., 2023; Zulkifli et al., 2022), research exploring its relation to oral health is limited. The few that have been undertaken have focused on the narratives and perspectives of parents and dental teams within dental settings (Alhammad et al., 2020; Junnarkar et al., 2023; Parry et al., 2023; Stein Duker et al., 2019). These studies illustrated challenges, with 52% of parents of autistic children reporting difficulties concerning three or more sensory variables, including (but not limited to) dentist drilling, bright lights and loud sounds, compared to 6% of parents of non-autistic children (Stein Duker et al., 2019) and how sensory sensitivities can impact the delivery of dental care services (Eades et al., 2019).

Our qualitative research deepens the existing understanding by offering a more detailed and nuanced contextual exploration of the unique experiences of autistic children themselve*s*. Through semi-structured interviews and adaptive data collection methods, the research demonstrates the range and idiosyncratic nature of oral health experiences encompassing aspects such as toothbrushing, dental visits and dietary habits. The findings reinforce the concept that there cannot be a uniform or ‘one-size-fits-all-all’ approach to oral healthcare for autistic children, but rather, individuality and diversity should be considered and embraced. Such good practice is a common finding across a wide range of health care settings (Babalola et al., 2024) and explains why traditional, generic, and universal messaging such as ‘you have to sit in the dental chair to get your teeth checked’ can be ineffective. A tailored, person-centred approach recognises and understands that each child’s journey is unique, and oral health support should be inclusive and adaptable to their needs.

The communication between dentists and autistic children and their parents is key to successful dental care (Erwin et al., 2022). The second theme, ‘Developing trust and routine through consistency, communication, and collaboration’, captured the multifaceted nature of autistic children’s oral health experiences. The analysis highlighted how trust could be formed through repeated interactions with the same dentist and transparent and consistent dialogue. This consistency not only helps alleviate uncertainty and worry but could also help children feel more comfortable accepting dental procedures. These underpinning principles should be tailored to the child’s individual needs. This emphasis on regularity and open communication resonates with existing literature, which has pointed to the challenges dental teams face in establishing and sustaining meaningful contact with autistic children (Eades et al., 2019; Erwin et al., 2022). Our research suggests a potential pathway to mitigating these challenges, as children expressed that clearly explaining the dental procedure, granting them autonomy and involving them actively in the dental visit processes can be key factors in facilitating a more comfortable and trustful dental experience. This aligns with wider healthcare studies where autistic individuals have emphasised the value of providing clear explanations (Maddox et al., 2020; Mason et al., 2021; Taghizadeh et al., 2019) and wider dental literature that recommends that autistic individuals should be included in decisions about their dental health (McMillion et al., 2022). Building on these, our study has captured the perspectives of those with lived experiences, particularly children, who are often overlooked in dental research. Such findings could also be applied to home-based oral health care routines, especially as parents are important collaborators in shaping and supporting their children’s oral health habits (Fenning et al., 2022). Parents could narrate the steps of toothbrushing while actively seeking the child’s input during the selection of toothbrushes/toothpaste and determining preferred settings and methods for toothbrushing. This is consistent with previous findings and underscores the importance of clear communication and active involvement with autistic people in enhancing their dental care experiences (Erwin et al., 2021). Such iterative communication process can help facilitate routines and encourage strategies closely aligned with the child’s individual preferences and sensory needs, promoting a collaborative and tailored approach to oral healthcare.

Finally, this research is novel in its efforts not only to learn from autistic children directly, but also to use more inclusive and accessible methods to access the views of children with a range of communication needs and preferences. By employing the Talking Mats^®^ method, children with limited verbal abilities or those typically reserved around unfamiliar adults were able to share their views on a range of oral health topics. For some, contrary to parental expectations, this evolved into a more detailed and nuanced conversation about their oral health experiences. Inclusive research methods are a key priority for innovations in the autism field (Happé & Frith, 2020; Nicolaidis, 2019), with many qualitative studies with autistic children relying solely on spoken interviews, which excludes a significant proportion of the population (Fayette & Bond, 2018; Nicholas et al., 2019). In addition, we have demonstrated that it is possible to integrate conventional and alternative communicative forms within one thematic analysis, including child-led communicative methods (e.g. choosing to draw or bringing personal objects). This study highlights the benefits of alternative communication methods such as Talking Mats^®^ and how these can be integrated within qualitative data collection methods to scaffold and enable communication (Hayden et al., 2024). This method is not solely a research tool but could be incorporated into oral healthcare discussions to support oral health habits. Dental professionals can actively collaborate with parents to enable the child’s voice to become a focal point, making dental visits more meaningful and inclusive.

### Implications

This study underscores the critical need for enhanced training and awareness among dental teams to better serve autistic children, focusing on their unique needs and their active participation. Essential to this is the partnership with children and their families, ensuring successful dental visits through a supportive and inclusive environment. This aligns with global health objectives, such as those advocated by the World Health Organization (2023), and Autistica’s 2030 Goals (Autistica, 2023), aiming for more accessible healthcare. Key training initiatives, including the Oliver McGowan programme (Health Education England, 2023) and WHO guidelines, provide a framework to address care disparities. Training should consider broad principles – such as trust-building, effective communication and consistency – alongside the flexibility to tailor care to individual children’s needs. This dual approach can enable dental professionals to apply universal principles while adapting to the distinct requirements of each autistic child and their family, promoting a personalised approach to dental care.

Our study highlighted that autistic children experience a wide range of sensory sensitivities, which can make dental environments particularly challenging. Dental professionals must be trained to recognise and mitigate these sensory issues. This includes creating a sensory-friendly dental clinic environment with reduced lighting and minimal noise. It is also important to acknowledge that sensory sensitivities are transdiagnostic, appearing across a range of neurodevelopmental and psychiatric conditions, including those related to anxiety, trauma and attachment (Callahan & Lim, 2018; van den Boogert et al., 2022). Recognising sensory sensitivities as a transdiagnostic phenomenon highlights the need for inclusive sensory intervention strategies beyond autism. Future research should explore sensory-based interventions across different clinical populations to ensure all children with sensory challenges can access appropriate support. Broadening the scope of research and practice will enable a more inclusive and supportive environment for all children facing sensory issues.

This research underscores parents’ pivotal role as primary facilitators in supporting optimal oral health habits for their children. Furthermore, it highlights the critical need for parents to have access to appropriate support and resources, thus empowering them to manage their children’s oral health needs confidently and effectively. Acknowledging the existing barriers within dental settings, including access to dentists generally, there is a need to broaden the scope of oral health training to other professionals who support families in across their child’s early-years. This approach ensures that every encounter with families is a potential opportunity to support parents with small approximations towards optimal oral health habits for their autistic child.

### Limitations

It is important to acknowledge a limitation in the study’s methodology. The foundational principles of the Talking Mats^®^ are based on the circles model, which requires a certain level of symbolic understanding for meaningful engagement (Mundt, 2021). Two children initially recruited into the study were unable to access these methods. While this exclusion highlights a gap in this research approach, it also points to the need for continued development of inclusive research methodologies that can accommodate a wider range of cognitive abilities (McKinney et al., 2021). The authors also acknowledge that Talking Mats^®^’ visual and structured format may have limited some children’s ability to express views not represented by the pre-determined symbols. Following the Talking Mats^®^ principles, children were encouraged to draw their own ‘symbols’. The researchers encouraged an open dialogue, adjusting their approach based on the children’s cues. The team proactively sought deeper insights when feasible, aiming to gather as detailed data as possible within the confines of the methodology employed.

In addition, our approach to data collection was guided by the principles of reflexive thematic analysis, with a particular focus on the depth and richness of the data. Consequently, we concluded data collection after recruiting 12 children (with data from 10 ultimately used), based on a careful and reflective judgement about the comprehensiveness of the developing themes, as well as practical constraints, including applicability within a wider project and funding. This approach aligns with the guidance of Braun and Clarke (2019) and O’Reilly and Parker (2012), who emphasise that the significance and robustness of qualitative findings derive from the interpretative process and the interconnectedness of themes, rather than the number of participants.

While we have included all available demographic information in Table 2, we acknowledge that the participants’ overall levels of language, adaptive, cognitive or social functioning was not captured. Although these additional details would have provided a broader context and a deeper understanding of the participants’ abilities, the information provided can still aid in the transferability of the study’s findings. While qualitative research does not aim to generalise findings across broad populations, we strive to provide rich, detailed insights into human behaviour and social interactions. Autism is indeed diverse, and the information gathered in this study may not be indicative of the entire population. However, by providing descriptions of our participants and contexts, we enable others to judge the applicability of our findings to their own settings. This approach enhances the transferability of our results, allowing them to be applied to other contexts based on similarities in circumstances and participant characteristics.

**Table 2.** Demographic details of the children interviewed.

	Pseudonyms	Age (years)	Gender	Ethnicity	Location of interview	Communication methods used
1	Summer	10	Female	White British	Home	Verbal plus Talking Mats^®^
2	Amber	11	Female	White British	Home	Mainly Talking Mats^®^ and use of toothbrushing props
3	Hannah	9	Female	White British	Home	Verbal plus Talking Mats^®^
4	Harry	10	Male	White British	Home	Mainly verbal
5	Ash	9	Non-binary	White British	Home	Verbal plus drawing and toothbrushing props
6	Ron	9	Male	White British	Home	Verbal plus Talking Mats^®^
7	Lennie	11	Male	White British	Home	Verbal plus Talking Mats^®^
8	Ellie	13	Female	White British	Home	Verbal plus Talking Mats^®^
9	Arun	7	Male	Pakistani/Asian British	School	Talking Mats^®^
10	Kai	7	Male	Any other Asian background	School	Mainly Talking Mats^®^
11	Collin (Excluded)	12	Male	White British	Home	N/A
12	Mia (Excluded)	8	Female	Pakistani/Asian British	Home	N/A

### Future directions

It has become increasingly clear that the development of an oral health support package (intervention) for families required not just an understanding of oral health, but also a deep insight into the lived experiences of autistic children. The findings from this study have informed the development of a new oral health intervention called toothPASTE (https://linktr.ee/toothpastestudy). This will be available in Autumn 2024 to all families and early-years professionals across health, education, and the third sector.

## Supplemental Material

sj-docx-1-aut-10.1177_13623613241288628 – Supplemental material for An ‘explosion in the mouth’: The oral health experiences of autistic children Supplemental material, sj-docx-1-aut-10.1177_13623613241288628 for An ‘explosion in the mouth’: The oral health experiences of autistic children by Amrit Chauhan, Kathy Leadbitter, Kara A Gray-Burrows, Karen Vinall-Collier, Nicola Pickles, Sarah R Baker, Zoe Marshman and Peter F Day in Autism

sj-docx-2-aut-10.1177_13623613241288628 – Supplemental material for An ‘explosion in the mouth’: The oral health experiences of autistic children Supplemental material, sj-docx-2-aut-10.1177_13623613241288628 for An ‘explosion in the mouth’: The oral health experiences of autistic children by Amrit Chauhan, Kathy Leadbitter, Kara A Gray-Burrows, Karen Vinall-Collier, Nicola Pickles, Sarah R Baker, Zoe Marshman and Peter F Day in Autism

sj-docx-3-aut-10.1177_13623613241288628 – Supplemental material for An ‘explosion in the mouth’: The oral health experiences of autistic children Supplemental material, sj-docx-3-aut-10.1177_13623613241288628 for An ‘explosion in the mouth’: The oral health experiences of autistic children by Amrit Chauhan, Kathy Leadbitter, Kara A Gray-Burrows, Karen Vinall-Collier, Nicola Pickles, Sarah R Baker, Zoe Marshman and Peter F Day in Autism
