# Supplementary material for: An ‘explosion in the mouth’: The oral health experiences of autistic children
Source: Autism. 2024 Nov 8;29(3):627–41. doi: 10.1177/13623613241288628 (PMC11894836; doi:10.1177/13623613241288628)
Supplement: sj-docx-1-aut-10.1177_13623613241288628 – Supplemental material for An ‘explosion in the mouth’: The oral health experiences of autistic children [file sj-docx-1-aut-10.1177_13623613241288628.docx]

An “explosion in the mouth”: The Oral Health Experiences of Autistic Children

**Supplementary file - Reflexivity**

The approach to reflexivity has been outlined to highlight that the author’s interpretations and analyses were continuously reviewed through the lens of their experiences, biases, and positions. Before her current role, AC had experience working closely with autistic children, working within the home, mainstream and special educational needs setting with verbal and non-verbal children. Her current position as a qualitative researcher, developing and evaluating complex oral health interventions offered additional experience and a different lens to her interpretive process. However, AC was mindful of the potential biases and preconceptions that this background might introduce, particularly in interpreting the experiences of the participants.

To ensure a reflective analysis, AC engaged in a debriefing process with the Principal Investigator (PD) following each interview. These discussions allowed for immediate reflection and discussion, allowing them to discuss initial interpretations and explore alternative perspectives. Furthermore, as part of their reflexivity practice, AC maintained a reflective journal, documenting thoughts, reactions, and emotions related to the data. These reflections helped identify and navigate potential biases during the analysis.

Furthermore, the authors were researchers from diverse disciplines, including Psychology, Dental public health, and Paediatric Dentistry. NP was a Patient and Public Involvement and Engagement (PPIE) co-applicant, who is a parent of an autistic boy and parent advocate for a local autism charity. Her expertise and background allowed a variety of viewpoints to be explored. Regular monthly meetings with the research team facilitated continual review of progress, including the analysis and refinement of emerging themes.

While member checking was not employed in this study, the involvement of a wider Patient and Public Involvement and Engagement (PPIE) panel was significant. The panel's review of the initial themes provided valuable insights from their lived experiences and perspectives, thereby helping to shape the final theme development.

Through self-examination, ongoing discussions with the principal investigator, contributions from a diverse research team, and feedback from the PPIE panel, the researchers aimed to ensure the credibility of their findings. These approaches ensured that the interpretations reflected the experiences of autistic children, informed by collective expertise and experiences.
