# Supplementary material for: An ‘explosion in the mouth’: The oral health experiences of autistic children
Source: Autism. 2024 Nov 8;29(3):627–41. doi: 10.1177/13623613241288628 (PMC11894836; doi:10.1177/13623613241288628)
Supplement: sj-docx-2-aut-10.1177_13623613241288628 – Supplemental material for An ‘explosion in the mouth’: The oral health experiences of autistic children [file sj-docx-2-aut-10.1177_13623613241288628.docx]

# Topic Guide

| Topic | Visual Scale | Option examples |
| --- | --- | --- |
| Things that I do (main starter mat) | Like/not sure/not like | Playing games, playing with trains, watching tv, listening to music, swimming, going to school, going to the dentist |
| What I use to brush my teeth | Like/not sure/not like | Electric, manual, three headed toothbrush, finger, silicon, bamboo, toothpaste, cloth |
| Where I brush my teeth | Like/not sure/not like | In the bath, next to the sink, in the bedroom, at school, watching tv, watching the tablet, in the kitchen |
| Who helps me brush my teeth (optional) | Like/not sure/not like | Mum, dad, child, teacher (school), grandma, brother, sister, grandad |
| Food I eat and drink | Like/not sure/not like | Crisps, bread, cake, custard, ketchup, jacket potato, biscuits, pizza, fish fingers, chicken nuggets, sweets/choc, Squash, water, coke, milk, milkshake, orange juice |
| Going to the dentist | Like/not sure/not like | Lights, waiting room, dentist, chair |

This topic guide outlines the topic, visual scale and option examples for conducting our interviews using Talking Mats®. Children are provided with various options related to a given topic (examples provided below) and asked to place these options along a visual scale. The questions posed are open-ended, such as "How do you feel about… playing games?" The children then position their responses on the mat, allowing them to visually express their feelings and opinions. Blank ‘options’ are provided to allow children to draw or express those that are not predefined.
