# Supplementary material for: An ‘explosion in the mouth’: The oral health experiences of autistic children
Source: Autism. 2024 Nov 8;29(3):627–41. doi: 10.1177/13623613241288628 (PMC11894836; doi:10.1177/13623613241288628)
Supplement: sj-docx-3-aut-10.1177_13623613241288628 – Supplemental material for An ‘explosion in the mouth’: The oral health experiences of autistic children [file sj-docx-3-aut-10.1177_13623613241288628.docx]

| Theme | Subtheme | Example codes (semantic and latent) |
| --- | --- | --- |
| The Diverse Sensory Nature of Oral Health Activities* | 1a. Toothpaste can feel like an “explosion in the mouth.” | Sensory overload, aversion to strong flavours, toothpaste texture issues, anticipatory discomfort/worry before using toothpaste, specific preferences to avoid sensory overload, preferences for type of toothpaste, metaphorical descriptions of sensory impact of toothpaste |
|  | 1b. ‘Too soft” or “too hard”: The importance of the right oral health tools and techniques | General dislike of toothbrushes, difficulty finding the right toothbrush, preferences for type of toothbrush, sensitivity to toothbrush bristle firmness, indicators of under-sensitivity/over-sensitivity in tool preference, “weird” feeling of dental tools on teeth, unexpected sensory experiences from dental equipment |
|  | 1c. An overwhelming environment | Sensitivity to noisy environments, discomfort with smells and noise while eating, intersection of sensory experiences, parallels between school and dental experiences, bright lights at the dentist contributing to discomfort |
| 2. Developing Trust and Routine through Consistency, Communication, and Collaboration | 2a. Navigating the uncertainty of dental care: Continuity and transparency | feeling more comfortable with the same dentist, unpredictability of who is in the dental clinic, familiarity with the dentist, lack of clear communication from dental professionals, invasion of space, wanting to know what’s going to happen (when brushing and going to the dentist), feeling better when the dentist talks through the procedure first- the need for clarity |
|  | 2b. Seeking autonomy and collaboration in oral health decisions | Wanting to be involved, ‘we’ as a collective, preference having options, making an engaging dental visit, wanting to choose own toothbrush and toothpaste, lack of communication with dentist, wanting to have control over ‘when’ and ‘how’ |
|  | 2c. Parental role and support in creating oral health habits | Parental reminders to brush teeth, parental regulation of eating habits, preference for parents to ‘take over’ brushing (active involvement), parental encouragement when brushing, dependence on parental prompts for brushing, parents as habit enforcers |
|  | 2d. Visual support for motivation and routine: Helpful vs. impractical | Visual reminders for brushing routines, digital distractions during oral health care, visuals are too disruptive or distractive, perception of visual tools as time-savers, moving on can be hard, practical challenges of using visual tools in the bathroom |

**Final coding output**

## **Iterative development of themes and codes**

The process of coding and theme development in reflexive thematic analysis is iterative, with codes and themes continually refined through repeated engagement with the data. For instance, the first theme was initially labelled “The Sensory Nature of Oral Health,” but was later developed to “The Diverse Sensory Nature of Oral Health Activities” after revisiting the data. This revision reflected a deeper understanding of the variability in participants' experiences. Researchers are encouraged to engage deeply with the data, allowing themes and codes to evolve in response to the complexities and nuances within the data. For example, the initial coding of the quote, ‘Because if the toothpaste is too minty, oh gosh. If the toothpaste is a little bit too minty, then I don’t like it,’ highlighted a general dislike of mint toothpaste, was "aversion to strong mint toothpaste." However, upon further examination of the dataset as a whole, we identified significant variability in how children described their aversion. Some participants described sensory overload from the strong flavours, while others specifically noted issues with the texture of the toothpaste. This deeper analysis also revealed anticipatory discomfort or worry before using toothpaste, with some children developing specific preferences to avoid using mint toothpaste. Consequently, more nuanced codes were developed, such as "sensory overload," "aversion to strong flavours," "toothpaste texture issues," and "anticipatory discomfort/worry before using toothpaste." This iterative and reflexive approach ensured that our coding more accurately captured the diverse and complex experiences of the participants, allowing us to develop a richer and more nuanced understanding of the data.
